# A systematic review of hot weather impacts on infant feeding practices in low-and middle-income countries

**DOI:** 10.3389/fped.2022.930348

**Published:** 2022-09-06

**Authors:** Jessica M. Edney, Sari Kovats, Veronique Filippi, Britt Nakstad

**Affiliations:** ^1^Centre on Climate Change and Planetary Health, Department of Public Health, Environments and Society, London School of Hygiene and Tropical Medicine, London, United Kingdom; ^2^Department of Infectious Disease Epidemiology, London School of Hygiene and Tropical Medicine, London, United Kingdom; ^3^Division of Paediatrics and Adolescence Medicine, Faculty of Medicine, Institute of Clinical Medicine, University of Oslo, Oslo, Norway; ^4^Department of Paediatrics and Adolescent Health, University of Botswana, Gaborone, Botswana

**Keywords:** breastfeeding, climate, infant care, heat stress, dehydration, systematic review

## Abstract

**Background:**

Increased rates of exclusive breastfeeding could significantly improve infant survival in low- and middle-income countries. There is a concern that increased hot weather due to climate change may increase rates of supplemental feeding due to infants requiring fluids, or the perception that infants are dehydrated.

**Objective:**

To understand how hot weather conditions may impact infant feeding practices by identifying and appraising evidence that exclusively breastfed infants can maintain hydration levels under hot weather conditions, and by examining available literature on infant feeding practices in hot weather.

**Methods:**

Systematic review of published studies that met inclusion criteria in MEDLINE, EMBASE, Global Health and Web of Science databases. The quality of included studies was appraised against predetermined criteria and relevant data extracted to produce a narrative synthesis of results.

**Results:**

Eighteen studies were identified. There is no evidence among studies of infant hydration that infants under the age of 6months require supplementary food or fluids in hot weather conditions. In some settings, healthcare providers and relatives continue to advise water supplementation in hot weather or during the warm seasons. Cultural practices, socio-economic status, and other locally specific factors also affect infant feeding practices and may be affected by weather and seasonal changes themselves.

**Conclusion:**

Interventions to discourage water/other fluid supplementation in breastfeeding infants below 6 months are needed, especially in low-middle income countries. Families and healthcare providers should be advised that exclusive breastfeeding (EBF) is recommended even in hot conditions.

## Introduction

The World Health Organization (WHO) recommends that infants are exclusively breastfed for the first 6 months of their life (1). Breast milk safely fulfills all nutritional and energy needs for the first few months of life and contains antibodies which are protective against childhood infections (2). A Lancet review of data from 153 countries found that exclusive breastfeeding (EBF) reduced infant mortality by 88% and was protective against infections and being overweight in later life and diabetes (2). EBF for the first 6months of life is one of the most effective interventions to promote adequate growth and prevent neonatal, infant, and childhood illness (3).

Infants in LMICs tend to experience longer durations of breastfeeding compared to those in high income countries (HICs) (2, 4). However, rates of early initiation of breastfeeding (the first hour after delivery is important for future breastmilk production) and EBF remain low in LMICs (2). Just over 40% of infants are exclusively breastfed until 6 months of age (with considerable variation in rates between settings) (5).

The initiation and duration of EBF can be determined by a range of factors including maternal employment, quality of care provided in health facilities, and local practices (6–8). Breastfeeding advice is provided by health workers, peers and family circles, and cultural beliefs can be highly influential on maternal intention to breastfeed exclusively (9–11). Past studies have detailed the common practice of giving supplementary water and/or tea to infants below the age of 6 months in many countries with hot climates (12, 13). Having or perceiving an inadequate milk supply is also associated with supplementing breastmilk with other foods or liquids (9, 14, 15). Another factor commonly identified with supplementing breast milk, particularly in hot climates, is a belief that the breastfeeding infant is thirsty, and therefore requires water (16–19).

The world is warming due to climate change. The International Panel on Climate Change (IPCC) estimates that global mean temperature increase will exceed 1.5–2°C by end of the century compared to the period 1850–1900 (20). Climate change is likely to increase impacts from more frequent and more intense heat waves. Observations confirm that the frequency and intensity of heatwaves has increased in Africa (21), while climate modeling has projected severe changes in both wet and dry extremes across Africa in the future (22).

This review is part of the CHAMNHA project that aims to understand how climate change may affect the health of pregnant women, newborns and infants in Africa (23). Despite increasing international attention on climate risks to maternal and neonatal health, no existing systematic review on heat impacts on infant feeding practices could be identified. Therefore, this review was conducted to explore how hot weather and/or climate conditions might affect perception of infant thirst and rates of non-EBF.

## Methods

### Objectives

To understand how hot weather conditions may impact infant feeding practices by identifying and appraising evidence that exclusively breastfed infants can maintain hydration levels under hot weather conditions, and by examining available literature on infant feeding practices in hot weather.

### Search strategy

Four medical science databases were searched: MEDLINE, EMBASE, and Global Health from Ovid, plus Web of Science. Search terms were scoped for the weather and climate exposures by researching existing systematic reviews involving ambient heat exposures, drought, and hot climates (24–26), and search terms for “infant feeding practices” were scoped by researching an existing systematic review of breastfeeding and other infant feeding practices (27).

See Table 1 for definitions used in this review.

**Table 1:** Definitions used in this review.

**Term**	**Definition**
Exclusive breastfeeding (EBF)	“No other food or drink, not even water, except breast milk (including milk expressed or from a wet nurse)” (1)
High ambient temperatures	Air temperatures of ≥30°C: the midpoint between the range of air temperatures (25–35°C) that the WHO recommends infants to be exposed to avoid hyperthermia and hypothermia (28)
High humidity	≥60% relative humidity: the upper limit to the United States Occupational Safety and Health Administration recommendation for relative humidity for indoor air (29)
Hot weather	Days where air temperatures exceed 30°C
Infant	A child up to the age of 12 months
Infant feeding practices	All methods of feeding infants food and water/other fluids, including exclusive breastfeeding, feeding infants expressed breastmilk or formula milk, feeding infants non-breast milk food and fluids, supplementing breast milk with water, tea, or other fluids, and supplementing breast milk with fluids and solid food
Low- middle-income country (LMIC)	As defined by the World Bank (30)
Newborn	An infant aged up to 28 days
Partial breastfeeding/mixed methods feeding	Feeding an infant breast milk and other food and/or fluids (excluding oral rehydration solution, drops and syrups for medical purposes) (1)
Prelacteal feeding	Giving a newborn food or drink prior to the first breast feed. It is not recommended by the WHO (31)

Inclusion and exclusion criteria for studies were defined prior to running the search in a PICO format:

- Population: infants up to the age of 1 year living in LMICs.
- Interventions/Exposures: ambient temperature, ambient humidity, hot weather, hot climate, hot season. Also, socio-cultural beliefs/medical advice relating to infant feeding in hot weather/climate conditions, EBF.
- Comparators: the same study sample under different weather/temperature conditions, the same study sample not breastfeeding exclusively, a control group from the same or very similar population.
- Outcome: weight loss, dehydration, infant feeding practices.

Articles were excluded if they:

- Reported heat exposures from heat sources unrelated to weather, unless heat exposures were controlled in order to simulate weather conditions.
- Referred to feeding practices of animals.
- Not in English.
- Were not published in peer-reviewed journals.
- Were reviews or commentaries.
- Examined feeding practices of children older than 1 year.
- Only report a study population in a high-income country.
- Were published prior to 1970.

The last search was conducted on 11th July 2020. The reference lists of included papers and review papers were examined for relevant articles. Google Scholar was also used to find more recent papers by searching for articles that cited those already identified. Search results were screened by one reviewer (JE) by title. Abstracts were then screened by the same reviewer and excluded if irrelevant. The full text versions of the remaining articles were downloaded and assessed against inclusion and exclusion criteria.

### Study quality

To appraise the quality of each article, the study designs for all articles were assessed according to predetermined quality criteria checklists adapted from the Joanna Briggs Institute critical appraisal tools for analytical cross-sectional studies (32), cohort studies (32), randomized controlled trials (33), and quasi-experimental studies (33) (for the purposes of this review, intervention studies that did not meet all of the methodological criteria of randomized controlled trials were designated quasi-experimental studies). These checklists were used to inform the rating of the quality of each study, which was either “poor,” “fair,” or “good” (details can be found in the **Tables 4** and **5**).

### Data extraction

For this review, data were extracted from each study on the study setting, population, sample size, methods, exposure measures, outcome measures, results, authors' conclusions, and potential sources of bias.

### Data synthesis

Findings were described using a narrative synthesis (34). Due to heterogeneity in study designs, a meta-analysis was not appropriate. The included studies were divided into two categories and findings compared within each group:

1. Studies that investigated the association between EBF and ambient temperature and/or humidity on infant hydration, including:
  i. Associations between EBF and indices of infant hydration (e.g., urine specific gravity, urine volume etc.).
  ii. Associations between temperature and indices of infant hydration;
  iii. Associations between EBF and indices of infant hydration modified by temperature.

2. Studies that investigated how weather factors influence infant feeding practices, including:
  i. Associations between ambient temperatures or the heat season and infant feeding practices;
  ii. Explanations for these associations, including reports whether infant water supplementation was perceived to be necessary in hot conditions.

For studies in the first group, descriptive data was summarized in tables (34). For studies in the second category, a thematic analysis of the key findings was conducted to identify pathways by which hot weather and climate exposures might impact infant feeding. Methodological robustness and study quality are described in the results and discussion.

## Results

**Figure 1:**
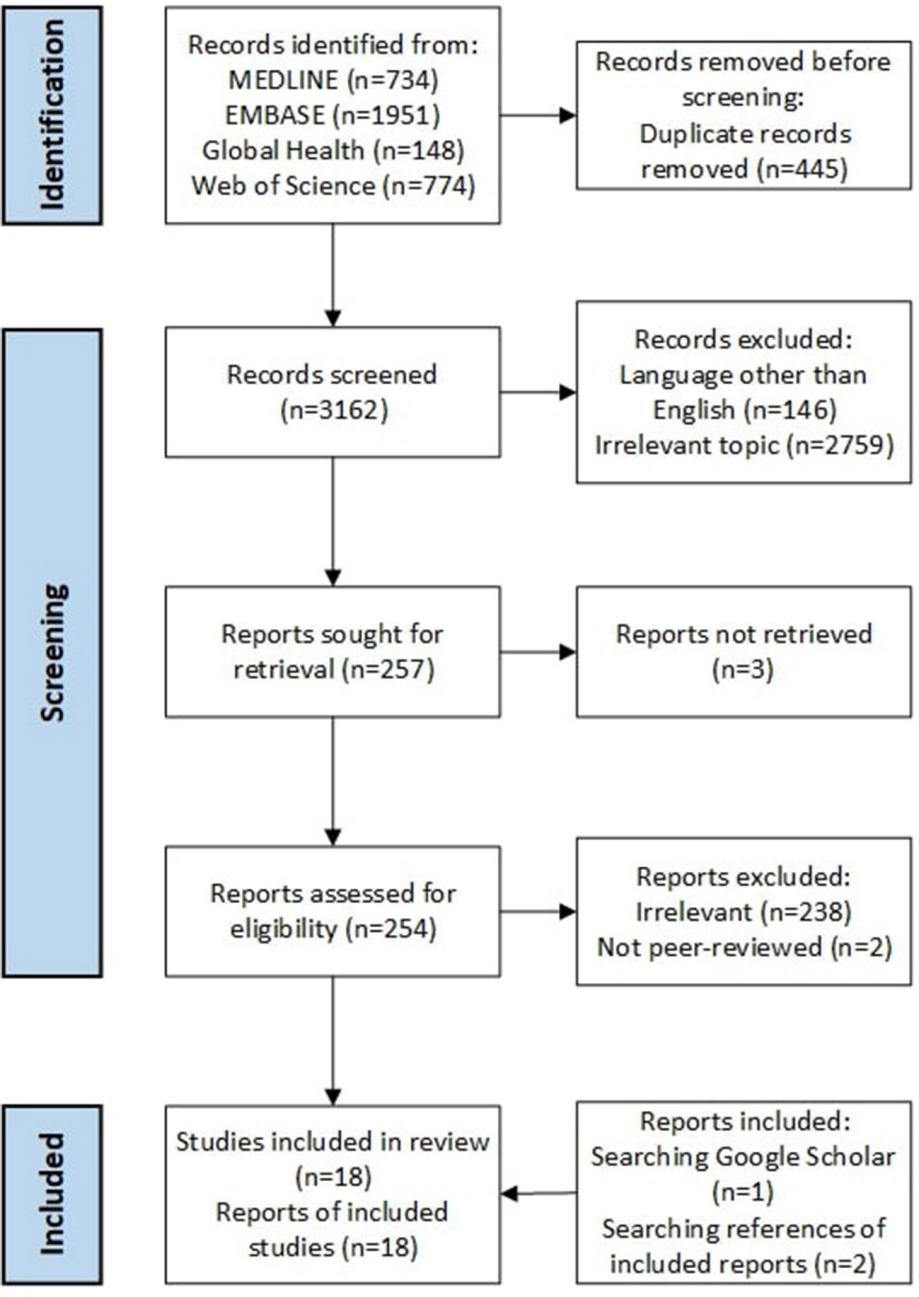
PRISMA flow diagram of included and excluded records [adapted from (35)].

Eighteen studies met the inclusion criteria. The full texts for three of these articles could not be located. Bibliography searching of already identified papers yielded two additional included articles and searching for articles which cited already selected papers in Google Scholar identified one additional article (see Figure 1).

Dates of publication ranged from 1978 to 2012 with most papers more than 10 years old. The review found a wide range of study designs:

1. Studies investigating the effect of EBF and ambient temperature and humidity on infant hydration (summarized in Table 2):
  ° Quasi-experimental studies (*n* = 6).
  ° Cross-sectional studies (*n* = 2).
  ° Cohort study (*n* = 1).

2. Studies investigating factors (specifically including weather/season/climate exposures) affecting infant feeding practices (summarized in Table 3):
  ° Cohort studies (*n* = 7).
  ° Cross-sectional study (*n* = 1).

**Table 2:** Summary of studies investigating infant hydration.

**Study authors (citation)**	**Location**	**Study subjects**	**Study design**	**Hydration measures and cut-offs**	**Results**	**Authors' findings**
Almroth and Bidinger (36)	India	Healthy infants aged 1–10 months (*n* = 44)	Quasi-experimental, using clinical measurements	USG and UO–cut-off values not reported	USG (of infants aged up to 6 months) = 1.004–1.036. UO = 66–1234 mml/l; mean = 1.011 (322 mml/l)	Under hot and dry conditions, healthy exclusively breast-fed infants do not require additional water.
Almroth (37)	Jamaica	Healthy EBF infants aged 2 weeks−4 months (*n* = 22)	Quasi-experimental, using clinical measurements	USG and UO–cut-off values not reported	USG = 1.005–1.015 (mean = 1.009). UO = 103–468 mml/l (mean = 258 mml/). Mean USG for individuals = 1.006–1.012 (UO = 139–358 mml/l).	Healthy, exclusively breast-fed infants can remain hydrated without supplementary water in a hot humid climate.
Armelini and Gonzalez (38)	Unknown “tropical” location	Healthy EBF infants aged 15–60 days (*n* = 8)	Cross-sectional, using clinical measurements	UO values below 200 mml/kg regarded as normal	Mean UO of samples collected at night = 137 mml/kg. Mean UO of samples collected in the afternoon = 171 mml/kg). Significant difference between means (*p* <0.01).	Urine osmalality was lower at night but did not exceed 200 mosmol/kg of water even in the hottest temperatures so supplementary water is likely not necessary for healthy exclusively breastfed infants in a hot and humid climate.
Ashraf et al. (39)	Pakistan	Healthy breastfed infants aged 2–4 months (*n* = 26)	Quasi-experimental, using clinical measurements	Water withheld for 8 days of study. Water given from day 8–15. DDAVP administered on day 15. USG values before and after the administration of DDAVP were compared. Haematocrit, sodium in breastmilk, and sodium in blood serum were compared at different stages to check for dehydration.	Significant increase in weight between day 1–8 and between day 8–15. No significant difference in haematocrit and serum sodium between day 8 and 1 and between day 15 and 8. No significant difference for USG between day 8 and 1. Significant increase in USG after infants received DDAVP.	Results indicate that the infants were not dehydrated when water was withheld and if needed, exclusively breastfed babies can concentrate urine when water is restricted
Brown et al. (40)	Peru	Breastfed infants aged <6 months (*n* = 40)	Quasi-experimental, using clinical measurements	Maximum USG 1.015 in the first few days of life, then 1.025–1.030 by 5 months	Standardized milk intakes = 4.0–12.1 gm/kg body weight per hour. Urine volume = 0.9–6.3 ml per kg body weight per hour. Max. USG in each infant 1.003–1.017. No associations between ambient temperature and urinary measures.	Healthy infants can maintain adequate hydration while exclusively breastfeeding in hot and humid conditions
Cohen et al. (41)	Honduras	EB LBW term male infants aged 0–4 months (*n* = 127)	Quasi-experimental, using clinical measurements	USG maximum below 1.018	Maximum USG = 1.001–1.012. USG associated with max. daily temperature only at 2 weeks of age. No association with humidity.	Results indicate that exclusively breastfed LBW infants do not require supplementary water, even under hot and humid conditions
Goldberg and Adams (42)	Unknown location in Sinai desert	Healthy EBF infants aged 40–150 days, from Bedouin villages (*n* = 15)	Cross-sectional, using clinical measurements	UO–cut-off values not reported	UO = 55–320 mmol/kg; mean = 164.5 mmol/kg	Supplementary water for breast-fed infants is not necessary to maintain hydration under hot and dry climatic conditions
Kusuma et al. (43)	India	Newborn infants with a gestational age of 350/7 weeks to 376/7 weeks born in a tertiary care hospital (n=205)	Cohort study, using a questionnaire and clinical measurements	Cumulative weight loss equal to or more than 10% was taken as ‘significant'. USG below 1.020 taken as normal.	No significant difference in weight loss or hypernatremia among the exclusively breastfed babies and partially breastfed babies	Near-term-born infants who are exclusively breastfed can maintain similar hydration status to that of PBF newborns in the first week after birth, even in summer months
Sachdev et al. (44)	Unknown “tropical” location	Healthy, male, exclusively breastfed babies aged 1–4 months attending a well-baby clinic (*n* = 45) AND doctors (*n* = 70) and nurses (*n* = 34) in the departments of pediatrics and obstetrics and gynecology in the same country	Quasi-experimental study using questionnaires, physical examinations, and clinical measurements	Breastmilk-intake, total fluid intake, urine output, UO, serum osmolality, weight change, rectal temperature. Cut-offs unclear. Compared study group (exclusively breastfed) with control group (partially breastfed)	Breastmilk intake and total fluid intake were significantly higher in the group that consumed only breastmilk compared to the control group after adjusting for confounders. No significant differences in urine output, urine or serum osmolality, weight change, or rectal temperature.	Exclusively breastfed babies don't require supplementary water in a tropical climate

**Table 3:** Summary of studies investigating determinants of infant feeding practices relating to hot seasons and high ambient temperatures.

**Study authors (citation)**	**Location**	**Study subjects**	**Study design**	**Results**	**Authors' explanations for associations**
Almroth et al. (45)	Lesotho	Mothers with children aged under 5; grandmothers of children aged under 5; women paid to take care of children aged under 5; local health professionals [Total unclear: 9 focus groups, individual interviews with 35 nurses & 8 mothers, 165 participants were surveyed in 1992, then 120 were surveyed in 1995 (unclear if these were different participants or not)]	Cross-sectional study, using interviews, questionnaires, focus groups	Breastfeeding common but EBF was an unknown concept. Mothers regularly gave infants water; nurses often identified as the source of advice for giving water. Hot weather was not cited as a reason for giving water.	Hot weather may not have been a factor in giving water because Lesotho rarely experiences very high ambient temperatures
Ashraf et al. (39, 46)	Pakistan	Mothers of newborn infants in Lahore before 1993 (*n* = 1,476)	Cohort study, interviews	EBF rare and negatively associated with age. Initiation of breastfeeding often delayed; prelacteal feeding the norm.	Reasons for associations not explored
Forman et al. (47)	Israel	Bedouin Arab mothers of apparently healthy newborn babies in the Negev in 1982	Cohort study, interviews, questionnaires	Month at which infants reached 2 months of age modified association between social support and feeding practices. Infants who turned 2 months old during the hot and dry season less likely to be EBF than infants turning 2 months in other seasons.	1. The month when infants reached two months old was independently associated with infant feeding practices–exclusive breastfeeding was less common at 2 months during the summer holidays (July/August), when mothers have a heavier workload with more childcare, and less support. 2. July/August are hot dry months with higher rates of diarrhoeal disease–illness in the family also reduces mothers' available time and increases workload
González-Chica et al. (48)	Brazil	Hospital-born children in 1982, 1993, and 2004 (periodically followed-up for 2 years) (*n* = 15,450)	Cohort, using interviews, clinical measurements	Season of birth & temperatures in 1st month of life associated with duration of breastfeeding (colder temperatures associated with shorter duration). Early intro of fruits and vegetables sometimes associated with lower environmental temperature in 1st month of life.	1. Reduced liquid intake on the part of the mother in colder seasons could affect infant feeding practices 2. Lack of central heating in homes may mean that mothers might be unwilling to get out of bed to breastfeed in a freezing cold home 3. Tendency to store bodily fat in the winter could be associated with decreased breast milk output. 4. Increased prevalence of infections and illnesses in colder months might prevent babies from suckling. Mastitis also more commonly diagnosed in colder months.
					5. Maternal education and socio-economic status modified the effect - poor people less likely to have central heating and less educated mothers might be more inclined to believe supplementing with water is advisable
Gray (49)	Kenya	Turkana mothers and their breastfeeding infants in 1987 −1989 (*n* = 101)	Cohort study, using interviews, clinical measurements, nutritional assessments, observation	Consumption of supplementary foods increased with less rainfall in infants below 4 months. In infants aged 4–9 months, consumption of milk and butterfat common, especially after wet months.	Mothers use milk and butterfat to supplement the fat lost from breastmilk during leaner months, when rainfall is higher
Hossain et al. (50)	Egypt	Singleton apparently healthy newborn children aged 0–47 weeks in 1987 (*n* = 152)	Cohort study, using interviews and questionnaires	Prelacteal feeding associated with warmer temperatures during the month of birth. Infants fed prelacteal food/drink less likely to be EBF in later months.	1. The association between prelacteal feeding and warmer temperatures may be due to birth attendant recommendations that babies are given water in summer months 2. Some mothers gave babies prelacteal drinks for perceived pharmacological effects
Jalil et al. (51)	Pakistan	Infants born in an urban slum in 1964–1978 (*n* = 910)	Cohort study, using clinical measurements and observations	50% of mothers introduced supplementary feeding within 1 month of birth. Among babies who didn't receive supplementary food, all received extra water during the hot season	1. Cultural belief that milk comes in on the third day prevents early breastfeeding initiation. 2. Colostrum thought stale, so discarded 3. Traditional practice of an important family member giving first (prelacteal) feed to imprint moral values upon them 4. Increase of feeding babies supplementary water in hot and dry season likely due to fears of dehydration
Naggan et al. (52)	Israel	Bedouin Arab mothers who delivered apparently healthy newborns in hospital or at home in 1981–1982 (*n* = 945)	Cohort study, using interviews, clinical measurements, clinical records	Babies born during wet cool months are EBF longer than babies born during the dry season.	Mothers may experience dehydration during the hot, dry season. There is no change in breast milk volume, but it becomes diluted so that enough nutrients are transferred to the baby. This may mean that they do not receive enough nutrients. This may be why exclusive breastfeeding occurs less in hot weather
Serdula et al. (53)	Egypt	Children aged between 6 and 35 months in 1978 and 1980	Cross-sectional study, using surveys	EBF (among infants aged 6–11 months) more prevalent in the hot season survey	1. During the hot season, pregnancy rates were higher, which resulted in less breastfeeding 2. Seasonal variation in breastfeeding may in part be due to women's need to work in agriculture at certain times of the year, which takes them away from their babies 3. Breastmilk output has been shown to decrease in the wet season in some studies. 4. Fears of diarrhoeal disease in their infants in the late summer months might have discouraged women from supplementing breastfeeding

Studies were conducted in Africa, Asia, and South America: Pakistan (*n* = 3), India (*n* = 2), Egypt (*n* = 2), Israel (*n* = 2), Brazil (*n* = 1), Honduras (*n* = 1), Lesotho (*n* = 1), Peru (*n* = 1), Kenya (*n* = 1), and Jamaica (*n* = 1). The country locations of three studies were not identified in the journal articles, but since these articles specified the locations were “tropical,” it was inferred that the settings were more likely to be in LMICs than in HICs.

### Effect of EBF on infant hydration in hot weather/climate conditions

The 6 quasi-experimental studies (see Table 2) involved requesting participating mothers to exclusively breastfeed their infants and investigating the effect of this on infant health and hydration status. Two studies were cross-sectional (38, 42) and a cohort study (43). The intervention group (in which mothers were requested to exclusively breastfeed) was compared to a control group (where mothers followed their usual feeding regime). It would not be possible to randomize for heat exposures. The studies were published between the 1970's and the 2000's. The most recent study was published in 2009 (43) but the findings of the quasi-experimental studies are likely to still be relevant.

Measures of infant hydration included urine specific gravity (USG), urine osmolality (UO), urine volume, total fluid intake, and infant weight change. USG and UO are two (correlated) measures of urine concentration and can be used to diagnose abnormal hydration levels when the subject's maximal urine concentration ability is known (54). There is no cut-off value for either USG or UO in the medical literature that distinguishes normal hydration from dehydration, as urine concentration ability is affected by many variables, including sex and age (55). In order to interpret USG or UO values, therefore, many studies also used additional health measures (e.g., identified participants' likely maximal urine concentration abilities) or compared with baseline values from start of study. Two studies used a cut-off value for “normal” USG; ≥ 1.020 (43) and ≥ 1.018 (41), but neither cited sources supporting these thresholds.

Nearly all the studies measured USG and/or UO and showed a high level of variance, reflecting the natural high variability in USG and UO levels, and also known variation in subgroups (infants of low birthweight and infants born near-term (with a gestational age of 35^0/7^−37^6/7^ weeks) (41, 43). In several studies, additional measures were also used to validate assigned outcome status or to investigate risk factors for the primary outcome, including USG, infant weight, fluid and/or breast milk intake, blood serum sodium, haematocrit, concentration of sodium in the mother's breast milk, rectal temperature, and blood serum osmolality. Increased haematocrit is a consequence of water loss and can therefore indicate dehydration (56–58). It is worth noting that there is no formal measurement methodology for dehydration in infants (59). Weight loss has traditionally been used as a “gold standard” measure of infant dehydration (59), but it is not always possible to obtain pre-dehydration weight for comparison (60, 61). A mix of measures has been shown to be most reliable for assessing dehydration (59–61).

As far as can be ascertained, temperatures and relative humidity levels measured during the studies ranged from 11–41°C to 10–96%, respectively. The average range of temperatures was 26.5–34.8°C and the average range of relative humidity levels was 38.8–69.2%. For the studies that recorded it, the average mean temperature was 29.7°C and the average mean level of relative humidity was 61%. Almost all studies that reported weather conditions reported mean temperatures above 30°C (36, 40–44) and most studies reported relative humidity to be 60% or above (37–41, 43).

All studies reported that even under hot conditions, exclusively breastfed infants maintained normal hydration levels without concentrating urine to maximal levels and concluded that supplementary water was therefore not necessary. Several studies concluded that exclusively breastfed infants could maintain normal levels of hydration in hot and humid conditions (37, 38, 41, 44). Only one study investigated hydration levels of exclusively breastfed infants in hot and dry conditions (42); these authors also concluded that the lack of water supplementation did not provoke abnormal dehydration. Furthermore, supplementary water did not appear necessary for exclusively breastfed infants that were low birthweight (41) or born near-term (43). No studies investigated or measured hydration levels or any other health indicators in the mothers.

Comparing exclusively breastfed and partially breastfed infants, no significant difference in incidence of weight loss or hypernatraemia was found (43), nor were there significant differences in urine output, urine or serum osmolality, weight change, or rectal temperature between exclusively breastfed infants and a control group (44). Even when infants who had been partially breastfed previously were breastfed exclusively for a week, observed levels of haematocrit and sodium in blood serum and USG did not change, suggesting that withholding water did not cause dehydration (39). In one study, infants were injected with desmopressin, which is a type of medication that limits the amount of water that is eliminated into urine (62). The infants then experienced a significant increase in USG, suggesting that exclusively breastfed babies can concentrate their urine when water is restricted and do not experience dehydration (39).

### Effects of weather and climate exposures on infant feeding practices

Most of the included studies of infant feeding practices in hot weather were cohort studies (see Table 3) (46–52); the remaining two studies had a cross-sectional design (45, 53).

Studies investigating how factors relating to the weather or season affect infant feeding practices reported very different strengths and directions of associations. A cohort study conducted in Brazil (48) and a cross-sectional study conducted in Egypt (53) found that prevalence and duration of EBF increased in the warmer months. Conversely, four cohort studies, all conducted in either Pakistan (46, 51) or Israel (47, 52), found the opposite - that prevalence and duration of EBF decreased in the warmer months. In Kenya, a cohort study found that rainfall was also associated with lower rates of EBF (49).

Proposed or investigated mechanisms behind these associations were varied. A cohort study conducted in Egypt (48) found that prelacteal feeding was associated with both warmer temperatures during the month of birth and with lower rates of EBF in later months of life. The authors proposed that birth attendants were advising mothers to give infants water, particularly in summer months. A cross-sectional study in Lesotho (45) reported advice from healthcare providers also encouraging mothers to feed their infants supplementary water, although hot weather was not the justification for this recommendation. Studies of communities in Pakistan (51) and Israel (46) reported beliefs among mothers that dehydration was a risk for their babies in the hot and dry season, and so supplemented their breast milk with water more frequently during these periods.

Some associations between weather and infant feeding practices were found to be modified or confounded by other variables. For example, a cohort study of Bedouin women in Israel found that the relationship between mothers' experience of social support and infant feeding practices was modified by the infant's season of birth (47). School holidays took place during the hot, dry months, placing more childcare responsibilities on breastfeeding mothers, therefore higher temperatures seemed to affect rates of EBF only by their coinciding with the end of the school term. Similarly, research in Egypt indicated that associations between hot weather and reduced rates of EBF could be in part explained by higher pregnancy rates in the hot season, rather than temperatures themselves (53). In some settings, breastfeeding while pregnant is believed to cause the breast milk to become poisonous, therefore mothers stop breastfeeding as soon as they become aware of pregnancy (17), although it is not clear from the report if this is the case in the Egyptian study population. It was also proposed that Egyptian women's participation in seasonal agriculture might take them away from their infants and limit breastfeeding, which could also explain why EBF was less prevalent in summer (53). Furthermore, lower rates of diarrhoeal disease occurred in the early summer months–women were less inclined to supplement breastfeeding when there was a high risk of diarrhoeal disease, for fear of giving infants contaminated water (53).

Some studies found associations in the opposite direction: reduced rates of EBF were associated with cooler or wetter weather. It was reported that in the leaner, wetter months, Kenyan Turkana mothers would feed infants milk and butterfat to supplement fat perceived to have been lost from breast milk (49). Studying Brazilian mothers, researchers proposed that reduced liquid intake on the part of the mother in colder seasons could cause real or perceived maternal dehydration, which might encourage supplementing breast milk with fluids to prevent dehydration transferring to the infant (48). Alternatively, the authors proposed that a lack of central heating in homes could mean that mothers were unwilling to get out of bed to breastfeed in a cold home, while increased prevalence of infections and illnesses in infants during colder months could prevent babies from suckling. These hypotheses have not been fully investigated by scientific means, however.

## Discussion

All studies investigating hydration in exclusively breastfed infants reported that even under hot conditions, exclusively breastfed infants maintained normal hydration levels, and concluded that supplementary water was therefore not necessary.

The methodological quality of most of these studies was fair (38–41) or poor (36, 37, 42) (see Tables 4, 5). Study designs often did not account for biases or confounding, which weakened the strength of their findings. Many also did not include a control group or any comparisons for the study infants, which meant that it was difficult to isolate any associations, or lack thereof, between EBF and dehydration in hot conditions. With all of this in mind, both of the studies rated “good” (43, 44) also concluded that exclusively breastfed infants do not require supplementary water or fluids in hot conditions, and their findings were found to be less susceptible to bias. Given this and that no study provided any evidence for increased risk of dehydration in exclusively breastfed babies in hot conditions, it would be reasonable to agree with the WHO and UNICEF guidelines that recommend healthy infants up to the age of 6 months are fed exclusively with breast milk, regardless of weather conditions.

**Table 4:** Methodological problems identified in included studies.

**Methodological problem**	**No. of studies**	**Study authors (citation)**
Unclear if participants included in any comparisons were similar (except in exposure status)	3	Almroth et al. (45); Ashraf et al. (39, 46); Serdula et al. (53)
Inclusion criteria for study subjects not defined	3	Almroth et al. (45); Armelini and Gonzalez (38); Goldberg and Adams (42)
Study subjects and setting not adequately described	5	Almroth (37); Almroth et al. (45); Armelini and Gonzalez (38); Cohen et al. (41); Goldberg and Adams (42)
Among intervention studies, true randomization was not used to assign participants to treatment groups	4 (out of 4 intervention studies)	Almroth (39); Almroth and Bidinger (40); Brown et al. (44); Sachdev et al. (37)
Among intervention studies, unclear if participants were blind to exposure group	4 (out of 4 intervention studies) – but impossible to blind participants given nature of intervention	Almroth (37); Almroth and Bidinger (36); Brown et al. (40); Sachdev et al. (44)
Outcome assessors were not blind to treatment assignment or to study hypothesis where control groups were not used	17	All studies but Forman et al. (47)
“Cause" and “effect” unclear in the study	2	Almroth et al. (45); Jalil et al. (51)
Unclear if exposures were measured in a consistent, valid, and reliable way	3	Almroth et al. (45); Armelini and Gonzalez (38); Serdula et al. (53)
Unclear if outcomes were measured in a consistent, valid, and reliable way	4	Armelini and Gonzalez (38); Ashraf et al. (39, 46); Forman et al. (47); Gray et al. (49)
Confounding factors not identified	10	Almroth (37); Almroth and Bidinger (36); Almroth et al. (45); Ashraf et al. (39, 46); Cohen et al. (41); Brown et al. (40); Goldberg and Adams (42)
Unclear if strategies to address confounding were used	10	Almroth (37); Almroth and Bidinger (36); Almroth et al. (45); Armelini and Gonzalez (38); Ashraf et al. (39, 46) ; Cohen et al. (41); Brown et al. (40); Goldberg and Adams (42)
Where applicable, no control group or control population studied	11 (out of 16 applicable studies)	Almroth (37); Almroth and Bidinger (36); Almroth et al. (45); Brown et al. (40); Cohen et al. (41); Forman et al. (47); Goldberg and Adams (42); Gray et al. (49); Hossain et al. (50); Jalil et al. (51); Naggan et al. (52)
Where applicable, follow-up time was incomplete, for which the reasons were not described or explored	5 (out of 15 applicable studies)	Almroth (37); Almroth and Bidinger (36); Almroth et al. (45); Cohen et al. (41); Hossain et al. (50)
Where applicable, unclear if strategies to address incomplete follow-up were utilized	10 (out of 15 applicable studies)	Almroth (37); Almroth and Bidinger (36); Almroth et al. (45); Armelini and Gonzalez (38); Ashraf et al. (39, 46); Brown et al. (40); Cohen et al. (41); Forman et al. (47); Hossain et al. (50); Sachdev et al. (44)
Unclear if appropriate statistical analysis was used	7	Almroth (37); Almroth and Bidinger (36); Almroth et al. (45); Armelini and Gonzalez (38); Ashraf et al. (39, 46); Cohen et al. (41); Brown et al. (40); González-Chica et al. (48); Goldberg and Adams (42); Gray et al. (49); Hossain et al. (50); Jalil (51)

**Table 5:** Critical appraisal of studies.

**Study authors (citation)**	**Rating**
González-Chica et al. (48)	Good
Hossain et al. (50)	Good
Kusuma et al. (43)	Good
Naggan et al. (52)	Good
Sachdev et al. (44)	Good
Armelini and Gonzalez (38)	Fair
Ashraf et al. (39, 46)	Fair
Brown et al. (40)	Fair
Cohen et al. (41)	Fair
Forman et al. (47)	Fair
Gray et al. (49)	Fair
Jalil et al. (51)	Fair
Serdula et al. (53)	Fair
Almroth (37)	Poor
Almroth and Bidinger (36)	Poor
Almroth et al. (45)	Poor
Ashraf et al. (39, 46)	Poor
Goldberg and Adams (42)	Poor

Studies investigating how factors relating to the weather, season, or climate affect infant feeding practices were largely rated “fair” (47, 49, 51, 53) or “good” (48, 50, 52) and reported a wide variety of findings. No conclusive general relationship between weather and/or seasonal changes and infant feeding practices could be ascertained–rather, relationships were often context specific, mediated by other distal factors.

Despite no evidence supporting the need (as established by this review), several studies investigating infant feeding practices in LMICs reported that mothers supplemented breast milk because they believed that their infants were at risk of dehydration in hot weather (49–51). This concern has been proposed elsewhere in the literature as a potential reason why women are not breastfeeding exclusively in many LMICs (17, 18, 63–68).

However, fear of infant dehydration in hot conditions is not the only mechanism by which weather can influence infant feeding practices. Time spent taking part in agricultural labor can vary according to weather conditions (69) and was identified in one of the included studies as potentially affecting breastfeeding duration (53). Similarly, a study in Ethiopia indicated that rainfall during the primary agricultural season in a child's first year of life had a significant impact on EBF duration due to mothers increased hours worked in farming (70).

While some of the studies included in this review (51, 52) found that grandmothers, aunts, or the mother's own beliefs influenced infant feeding practices, others (44, 45, 50) reported that it was healthcare providers who advised mothers to give infants below the age of six months supplementary water. Similar findings have been reported elsewhere in the literature (64, 68)–they were not included as studies in this systematic review as they did not match the inclusion criteria. Healthcare providers have an important role to play in encouraging optimal breastfeeding, being one of the main sources of breastfeeding advice and knowledge in many communities. A study of infant feeding practices in Tanzania found that women who received advice on EBF were five times more likely to breastfeed exclusively (18). In communities that are both more vulnerable to climate risks and where access to formal healthcare is limited, increasing heatwaves could increase concerns regarding infant hydration.

### Limitations

Only one reviewer screened, selected, assessed, and summarized the included studies, which means that these stages of the review process may have been affected by bias. However, the other co-authors were consulted during these processes to reduce bias. Secondly, this review included studies published as early as 1978, due to the limited number of available studies matching the inclusion criteria. There may be a higher chance of such studies containing outdated information or knowledge, or their findings being less relevant to current practices and/or trends. This review also only included studies published in English, with relevant studies published in other languages probably missed. The literature search was not conducted in regional databases such as Africa-wide, which may have also led to missed records. Other limitations include the lack of detail in included studies on hot weather exposures (so it was not possible to directly compare temperature exposures) and lack of information on the hydration status of the mother. It is reasonable to assume that the mothers in studies were well-hydrated but, in some settings, mothers may be dehydrated with poor nutrition.

## Conclusion

A systematic review of studies investigating the ability of exclusively breastfed infants to stay hydrated in hot weather/climate conditions was conducted and found no evidence that exclusively breastfed infants required additional water or other liquids. A systematic review investigating hot weather and climate impacts on infant feeding practices in low- and middle-income settings was also conducted and found that multiple pathways exist by which temperatures and weather may influence infant feeding practices, including the belief that infants required water and/or other liquids alongside breast milk in hot weather or seasons. In addition, some factor, such as the demands on a woman's time (work or childcare), are highly seasonal and/or weather-dependent and linked to reduced time spent breastfeeding.

Global average temperatures are increasing, which means that populations everywhere will be exposed to higher daily temperatures. It has also been suggested that as LMICs economically transition, overall breastfeeding rates could decrease to similar levels to those of HICs (1). It is a concern that there has been a lack of studies conducted in the last two decades on infant feeding practices in hot weather. Future studies of infant feeding practices should use the WHO definition of ‘EBF' consistently, to improve the validity and compatibility of results. Many countries are already experiencing effects of climate change, so research in the worst-affected areas could be particularly relevant for determining intervention priorities for infant health as climate change progresses.

## Data availability statement

The original contributions presented in the study are included in the article/supplementary material, further inquiries can be directed to the corresponding author.

## Author contributions

JE undertook the screening and analyses and led on the drafting of the article. VF, SK, and BN contributed to the review designed and contributed to the drafting of the article. All authors contributed to the article and approved the submitted version.

## Funding

This work was supported by the Natural Environment Research Council (NERC) [Grant Numbers NE/T013613/1, NE/T01363X/1]; Research Council of Norway (RCN) [Grant Number 312601]; The Swedish Research Council for Health, Working Life and Welfare in collaboration with the Swedish Research Council (Forte) [Grant Number 2019-01570]; and the National Science Foundation (NSF) [Grant Number ICER-2028598]; coordinated through a Belmont Forum partnership.

## Conflict of interest

The authors declare that the research was conducted in the absence of any commercial or financial relationships that could be construed as a potential conflict of interest.

## Publisher's note

All claims expressed in this article are solely those of the authors and do not necessarily represent those of their affiliated organizations, or those of the publisher, the editors and the reviewers. Any product that may be evaluated in this article, or claim that may be made by its manufacturer, is not guaranteed or endorsed by the publisher.
